# First magnetic particle imaging to assess pulmonary vascular leakage in vivo in the acutely injured and fibrotic lung

**DOI:** 10.1002/btm2.10626

**Published:** 2023-11-29

**Authors:** Xin Feng, Pengli Gao, Yabin Li, Hui Hui, Jingying Jiang, Fei Xie, Jie Tian

**Affiliations:** ^1^ CAS Key Laboratory of Molecular Imaging, Beijing Key Laboratory of Molecular Imaging Institute of Automation, Chinese Academy of Sciences Beijing China; ^2^ School of Artificial Intelligence, University of Chinese Academy of Sciences Beijing China; ^3^ School of Biological Science and Medicine Engineering & School of Engineering Medicine, Beihang University Beijing China; ^4^ Key Laboratory of Big Data‐Based Precision Medicine (Beihang University) Ministry of Industry and Information Technology Beijing China; ^5^ School of Engineering Medicine, Beihang University Beijing China; ^6^ College of Pulmonary and Critical Care Medicine, Chinese PLA General Hospital Beijing China

**Keywords:** in vivo, lung injury, magnetic particle imaging, quantitative, vascular leakage

## Abstract

Increased pulmonary vascular permeability is a characteristic feature of lung injury. However, there are no established methods that allow the three‐dimensional visualization and quantification of pulmonary vascular permeability in vivo. Evans blue extravasation test and total protein test of bronchoalveolar lavage fluid (BALF) are permeability assays commonly used in research settings. However, they lack the ability to identify the spatial and temporal heterogeneity of endothelial barrier disruption, which is typical in lung injuries. Magnetic resonance (MR) and near‐infrared (NIR) imaging have been proposed to image pulmonary permeability, but suffer from limited sensitivity and penetration depth, respectively. In this study, we report the first use of magnetic particle imaging (MPI) to assess pulmonary vascular leakage noninvasively in vivo in mice. A dextran‐coated superparamagnetic iron oxide (SPIO), synomag®, was employed as the imaging tracer, and pulmonary SPIO extravasation was imaged and quantified to evaluate the vascular leakage. Animal models of acute lung injury and pulmonary fibrosis (PF) were used to validate the proposed method. MPI sensitively detected the SPIO extravasation in both acutely injured and fibrotic lungs in vivo, which was confirmed by ex vivo imaging and Prussian blue staining. Moreover, 3D MPI illustrated the spatial heterogeneity of vascular leakage, which correlated well with CT findings. Based on the in vivo 3D MPI images, we defined the SPIO extravasation index (SEI) to quantify the vascular leakage. A significant increase in SEI was observed in the injured lungs, in consistent with the results obtained via ex vivo permeability assays. Overall, our results demonstrate that 3D quantitative MPI serves as a useful tool to examine pulmonary vascular integrity in vivo, which shows promise for future clinical translation.

**Translational Impact Statement**

This study demonstrates the feasibility of using 3D MPI to sensitively visualize SPIO extravasation to the acutely injured and fibrotic lung in living animals, thus providing a noninvasive and quantitative tool to assess pulmonary vascular leakage in vivo. 3D MPI shows great promise for pulmonary research and future clinical translation.

## 1 INTRODUCTION

Healthy human lung contains approximately 400–700 million alveoli, and each one of them is surrounded by numerous capillaries that cover 70% of its surface area.^1^ Under normal conditions, pulmonary endothelium forms a tight barrier between the blood and interstitium of the lung, preventing the passage of liquid and macromolecules from the blood to the tissue. During acute lung injury and chronic lung diseases, this barrier is often disrupted, resulting in extravasation of blood constituents into the lung interstitium and breakdown of homeostasis.^2^, ^3^ Increased vascular permeability has been reported to significantly contribute to mortality in acute respiratory distress syndrome^2^, ^4^, ^5^ and is related to poor prognosis in pulmonary fibrosis (PF).^6^, ^7^, ^8^ Indeed, the vasculature plays an active role in tissue repair and fibrosis. However, the temporal and spatial heterogeneity of endothelial barrier disruption during diseases progression remains to be elucidated. As this topic has received increasing attention, there is a need to develop techniques that can visualize and quantify the changes in pulmonary vascular integrity in vivo over the course of a disease.

Currently in research settings, pulmonary vascular permeability assessment depends on terminal assays, such as the Evans Blue extravasation test and the bronchoalveolar lavage fluid (BALF) total protein test.^9^ However, these assays cannot track dynamic permeability changes in the same animal and cannot reveal the spatial distribution of vascular disruption. Although these assays can provide quantitative assessment of the permeability, they treat the entire lung as a whole and generate averaged readouts that are not informative enough. Tissue injury and endothelial dysfunction are often change with space and time in many diseases, such as PF and lung cancer.^10^, ^11^ The inability to evaluate the heterogeneity of vascular disruption may hinder the research on causality and reciprocity between endothelial dysregulation and disease progression.

In vivo imaging is an important tool for the study of dynamic biological processes. CT imaging is regularly used in clinics and pulmonary research to identify structural abnormalities and pulmonary edema.^12^, ^13^, ^14^ However, it is unable to differentiate hydrostatic pressure edema from permeability edema, where only the latter is associated with vascular permeability. Currently, there is a lack of in vivo imaging techniques that specifically target pulmonary vascular disruption. NIR fluorescence imaging and gadofosveset‐based MR imaging have shown some promise in this regard but have so far only been reported by a few research groups.^11^, ^15^

Magnetic particle imaging (MPI) is an emerging tracer‐based in vivo imaging modality that shows great promise in biomedical research and clinical translation. It uses superparamagnetic iron oxide particles (SPIOs), a radiation‐free contrast agent widely used in MR, as tracers to generate tomographic images with sensitivity comparable to positron emission tomography (PET). MPI signal is not attenuated by biological tissues, thus enabling deep tissue and whole‐body imaging in vivo. Currently, MPI has been used in a variety of biomedical applications, including long‐term cell tracking,^16^ tumor molecular imaging,^17^ functional neuroimaging,^18^ and cardiovascular imaging.^19^ MPI is particularly suitable for pulmonary imaging^20^, ^21^, ^22^ because its image quality is not affected by the alveolar air–tissue interface, which is a major challenge for MR and ultrasound imaging. MPI has been successfully applied to pulmonary perfusion imaging,^20^ ventilation imaging,^21^ and inhaled aerosol tracking.^22^

In this study, we established an in vivo imaging method based on 3D MPI to visualize and quantify pulmonary vascular leakage. The proposed imaging technique was validated in acute lung injury and PF mouse models, and in vivo imaging results correlated well with those obtained via histopathology and ex vivo permeability assays. To the best of our knowledge, this is the first use of MPI to assess pulmonary vascular permeability. It adds to the previous work on MPI pulmonary imaging and offers a novel in vivo permeability assay for pulmonary research, which shows promise for future clinical translation.

## 2 RESULTS

### 2.1 Characterization and MPI imaging of synomag®

**FIGURE 1:**
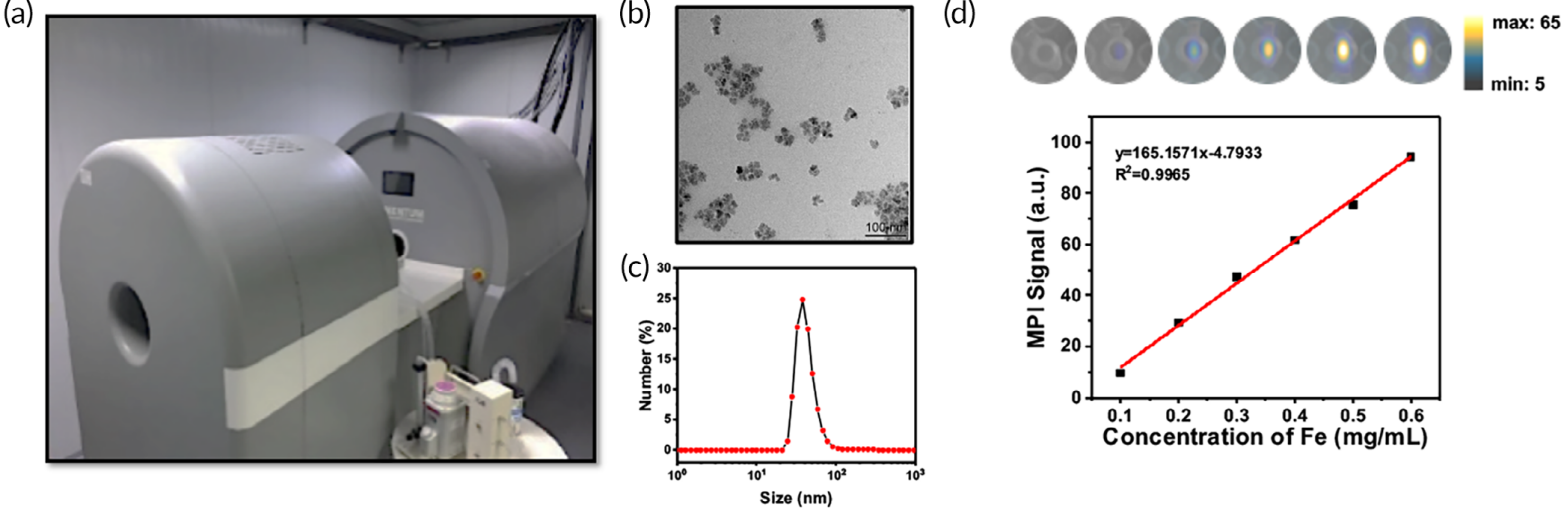
MPI system and the characterization of the dextran coated SPIO synomag®. (a) Magnetic insight momentum MPI scanner and the custom‐built CT scanner. (b) TEM image of synomag®. (c) DSL hydrodynamic size distribution of synomag®. (d) Linearity between synomag® concentration and MPI signal.

In this study, MPI imaging was conducted on the small‐animal MOMENTUM scanner (Magnetic Insight, CA, USA, Figure 1a) and a dextran coated SPIO synomag® was used as the imaging tracer.^23^ Synomags are nanoflower‐shaped particles with an average core size of approximately 20 nm, as revealed by TEM (Figure 1b). The hydrodynamic size of synomag® was measured to be 140.6 nm with a PDI of 0.41 (Figure 1c). Previous studies have shown that nanoparticles with hydrodynamic sizes >30 nm have negligible penetration through the endothelium.^22^ In disease states in which the blood–lung barrier is disrupted, circulating nanoparticles can pass through the endothelium and extravasate into the alveolar space. Therefore, we hypothesized that synomag® will only leak into the lung tissue where the endothelial barrier is disrupted. To investigate the feasibility of using MPI signal to quantify the extravasation of synomag®, the linearity between MPI signal and the tracer concentration was investigated. As shown in Figure 1d, the MPI signal intensity correlated linearly with the iron concentration (*R* ^2^ = 0.9965), highlighting its potential for quantitative imaging.

### 2.2 3D MPI visualizes the pulmonary vascular leakage in vivo in the OA‐mediated acute lung injury model

The feasibility of using 3D MPI to visualize pulmonary vascular leakage in vivo was initially demonstrated in an OA‐medicated lung injury model, which is well‐known for consistently inducing vascular permeability change.^24^ On the day of imaging, the mice were randomly separated into the OA and control (CTR) groups. Mice in the OA group were intravenously injected with 0.1 mL/kg of OA, and the CTR group was injected with the same volume of vehicle. Immediately after OA administration, typical acute respiratory distress syndromes (ARDS) such as shortness of breath and rapid heart rate were observed. ARDS was confirmed by percutaneous SpO_2_ measurements. The SpO_2_ of the mice in the OA group was 93.8 ± 1.0% compared to 96.7 ± 0.5% for the CTR group.

**FIGURE 2:**
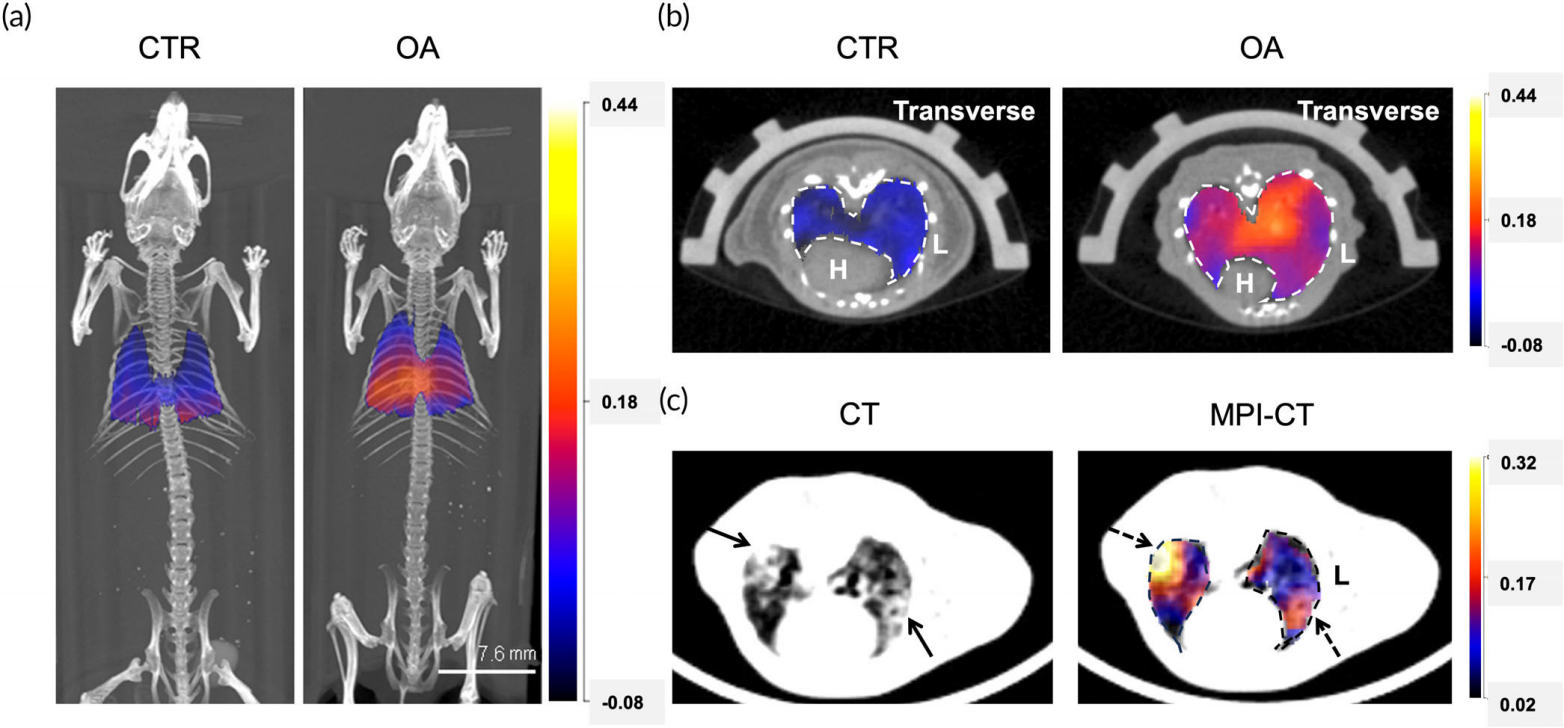
In vivo 3D MPI‐CT images visualizing the extravasation of synomag® into the acutely injured lung. (a) Representative 3D MPI‐CT images of the OA‐treated mice (OA) and the controls (CTR); MPI and CT images were presented in color and grayscale, respectively. (b) Representative transverse 3D MPI‐CT cross‐sections of the OA and CTR mice; dashed lines outline the lung region; L, lung; H, heart. (c) CT and MPI‐CT cross‐sections of an injured lung; black solid arrows: pulmonary edema, black dashed arrows: vascular leakage.

Once ARDS was established, 3 mg Fe/kg of synomag® was injected into the tail vein. At 8 h post‐tracer administration, mice were anesthetized and imaged with MPI and CT in vivo. MPI projection images were reconstructed and co‐registered with the CT image to obtain anatomical information. Lung segmentation was performed based on the CT image. Representative 3D MPI‐CT images and animations are shown in Figure 2a and Video S1 (http://www.mpilab.net/owncloud/index.php/s/qseqX7m9ezVvrua), where MPI and CT images were presented in color and grayscale, respectively. As demonstrated in Figure 2a, the pulmonary MPI signal of the healthy mice was close to 0, demonstrating the clearance of synomag® from circulation at the time of imaging. On the contrary, pulmonary MPI signal of the OA‐treated mice was markedly increased, suggesting the extravasation of synomag® into the extravascular space due to blood–lung barrier disruption. Moreover, the transverse cross‐section of MPI revealed a diffuse yet inhomogeneous signal distribution (Figure 2b), suggesting the heterogeneity of tissue injury and vascular leakage. To verify that the inhomogeneity of MPI signal is indeed resulting from heterogenous tissue injury, cross‐sectional CT, and MPI‐CT images were compared and presented in Figure 2c. CT image highlights patchy areas with pulmonary edema, as pointed out by the black arrows. Intriguingly, enhanced MPI signal can be observed from the same areas (black dashed arrows). Therefore, MPI identifies pulmonary regions with severe vascular leakage, which correlated well with the pulmonary edema observed in CT.

**FIGURE 3:**
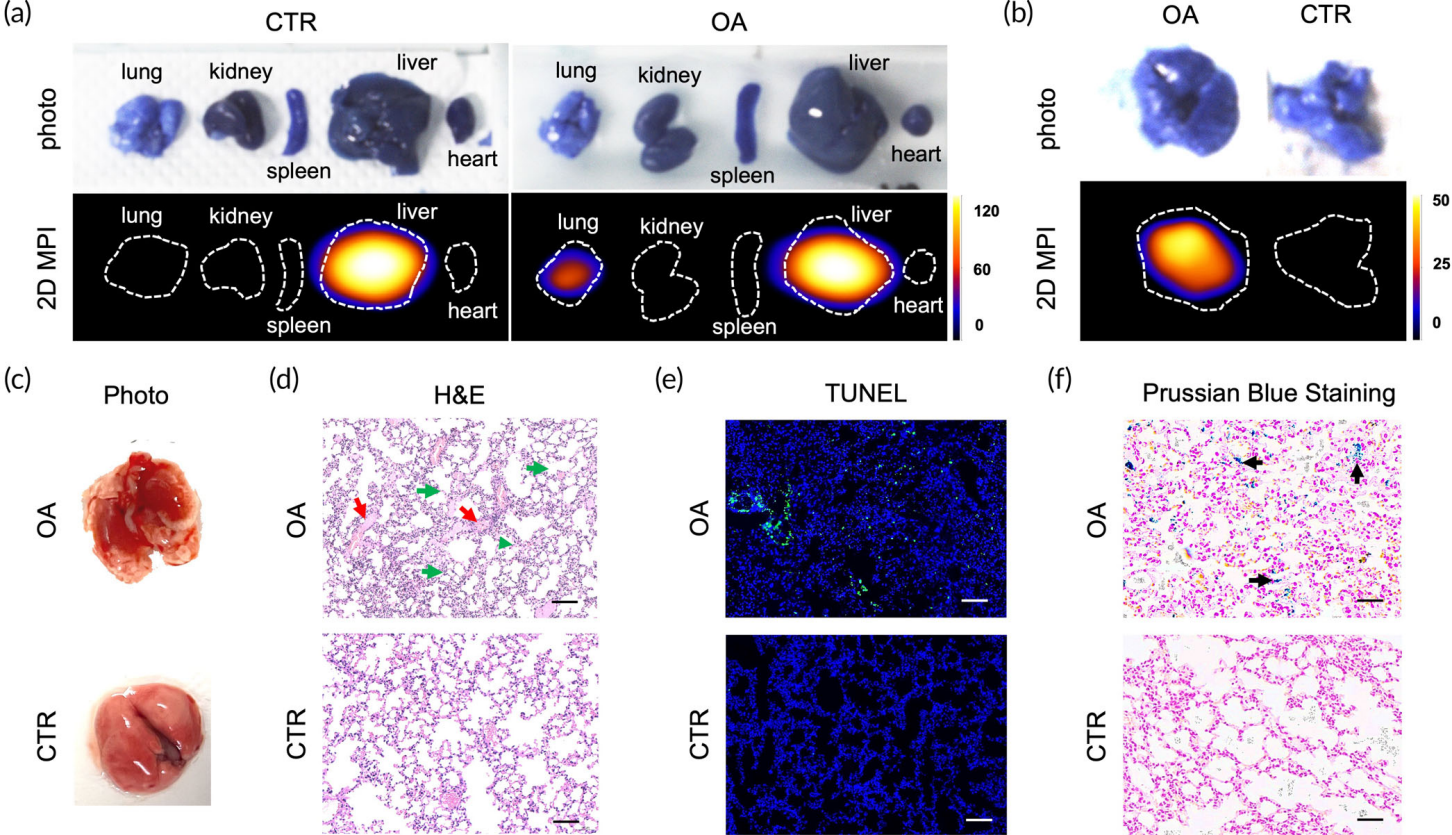
Ex vivo 2D MPI images and histopathological results. (a) Representative 2D MPI images of major organs excised from OA‐treated mice (OA) and the controls (CTR). (b) Lungs in (a) imaged side‐by‐side. (c) Digital photo of the lungs excised from the OA and CTR mice. (d) H&E histology of the healthy and injured lungs; red arrows: vascular leakage, green arrows: extrusion of neutrophils. (e) TUNEL staining of apoptotic cells (green). (f) Prussian blue staining of extravasated synomag® (blue stained particles, black arrows) in the injured lung. Bar = 100 μm.

To validate the in vivo imaging findings, the mice were sacrificed at 8 h, and major organs, including the lung, kidney, spleen, liver, and heart were harvested and imaged with 2D MPI. Representative ex vivo MPI images of organs are shown in Figure 3a. In the CTR group, MPI signals were observed primarily in the liver, but not in the lung or heart, confirming that the tracer had been cleared from the circulation and accumulated to liver at this time point. In contrast, a signal was observed in the lungs of OA‐treated mice. To verify and better illustrate the pulmonary signal difference, the lungs of OA‐treated mice and healthy mice were imaged side‐by‐side (Figure 3b). Indeed, lungs of OA‐treated mice exhibited significantly higher MPI signal as compared to the controls, agreeing with the in vivo imaging results.

To further verify the tissue injury and extravasation of SPIOs, histopathological evaluations of the excised lungs were performed. As shown in Figure 3c, the lungs of the OA‐treated mice appeared red with micro‐bleeding, compared to the homogenous pink color of the healthy lungs. Hematoxylin and eosin (H&E) histology of the lungs from the OA group showed diffuse alveolar damage, including thickening of the alveolar wall, infiltration of neutrophils, and diffuse hemorrhage (Figure 3d). TUNEL staining revealed an increased number of apoptotic cells (green) in the OA‐treated lungs (Figure 3e). Prussian blue staining highlighted SPIOs as blue‐stained particles, and SPIO extravasation in the interstitial and alveolar spaces was obvious in the OA‐treated lungs (Figure 3f). Overall, ex vivo histopathology validated the establishment of acute lung injury and confirmed that the increased MPI signal was registered from the extruded SPIOs in the lung tissue.

### 2.3 In vivo 3D MPI quantification of pulmonary vascular leakage

**FIGURE 4:**
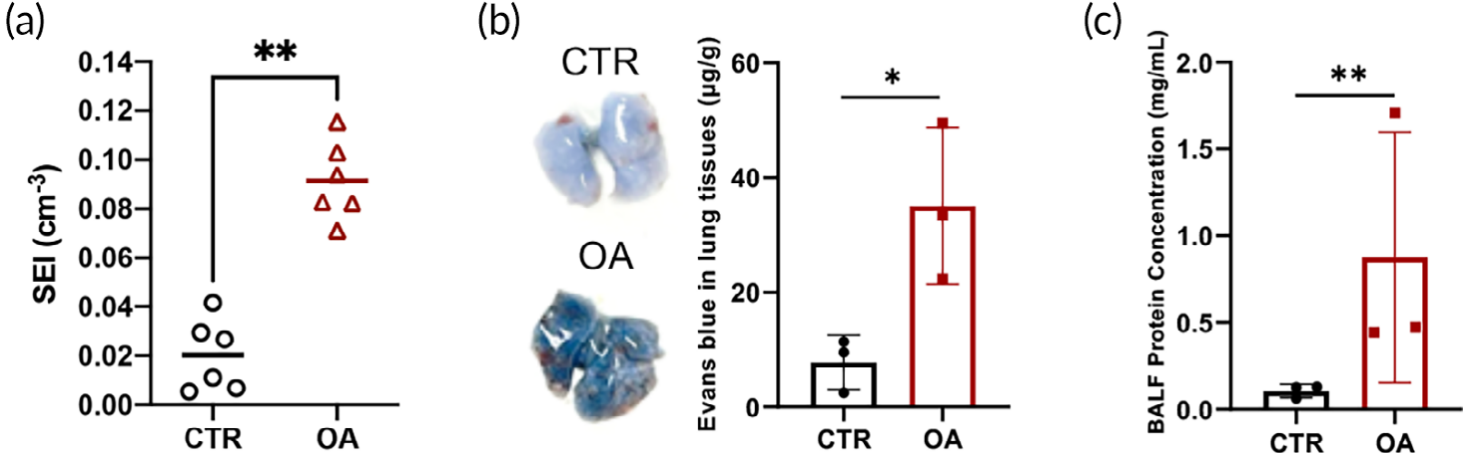
Quantitative assessment of pulmonary vascular leakage via in vivo 3D MPI, Evans blue extravasation assay, and BALF protein concentration test. (a) SEI of OA‐treated and control mice quantified from in vivo 3D MPI images (*n* = 6). (b) Evans blue vascular permeability assay reveals increased dye extravasation in the injured lungs (*n* = 3). (c) BALF from the OA‐treated mice contains significantly higher protein concentration as compared to the controls (*n* = 3). *p < 0.05, **p < 0.01.

Based on the in vivo 3D MPI images, we defined the SPIO extravasation index (SEI) to quantify the pulmonary vascular leakage. The SEI was calculated as the signal ratio between the lung and the entire body and normalized to the total volume of the lung. The SEI characterizes the fraction of injected SPIOs that extravasates into the unit lung volume. As shown in Figure 4a, the average SEI of the OA group (0.091 ± 0.016 cm^−3^) was approximately 4.5 times higher than that of the CTR group (0.020 ± 0.015 cm^−3^), and the difference was statistically significant (*p* < 0.01).

To validate the quantitative imaging results, permeability assays including the Evans blue extravasation test and BALF protein concentration test were employed. As shown in Figure 4b, 30 min after Evans blue injection, the lungs of the OA‐treated mice were clearly blue compared to the healthy lungs. The average uptake of Evans blue increased to 35.09 ± 13.70 μg/g in the lungs of the OA‐treated mice, compared to 7.79 ± 4.71 μg/g in the lungs of the controls (*p* < 0.05). The BALF protein concentrations were 0.87 ± 0.72 and 0.11 ± 0.04 mg/mL for the OA‐treated and healthy mice, respectively, with a statistically significant difference (*p* < 0.01). Overall, these results verified the enhanced pulmonary vascular permeability in the OA‐treated mice, confirming the correlation between the results obtained from the in vivo imaging in living animals and the ex vivo terminal assays.

### 2.4 Increased pulmonary vascular permeability in the PF mouse model induced by bleomycin

**FIGURE 5:**
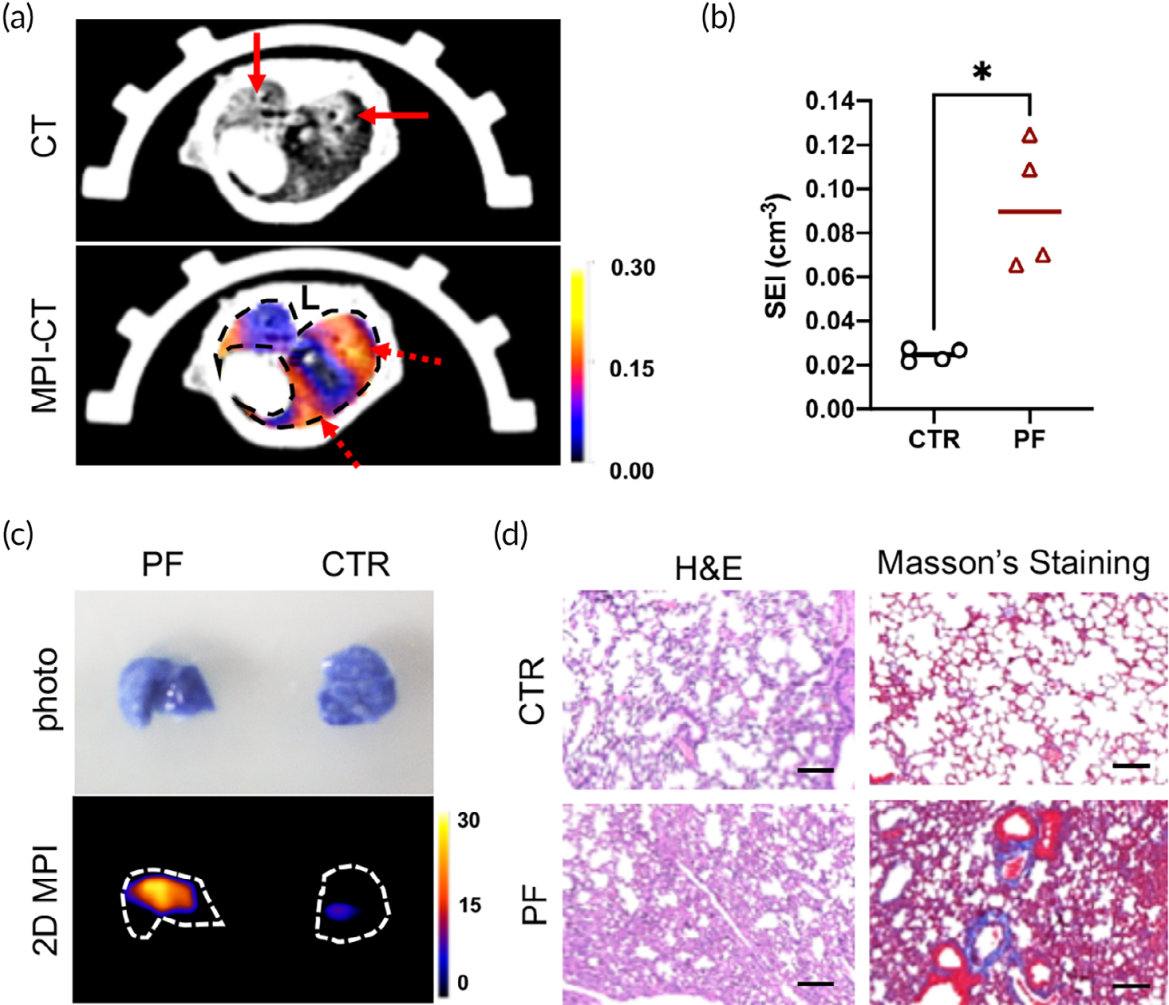
In vivo 3D MPI‐CT cross‐sections, in vivo image quantification and histopathological results of the fibrotic mouse lung. (a) Representative CT and MPI‐CT cross‐sectional images of the PF mice; red solid arrows: fibrotic regions in the CT image; red dashed arrows: regions with hyperpermeable vessels in the MPI image. (b) In vivo SEI of the CTR and PF mice (*n* = 4), *p < 0.05. (c) Representative 2D MPI images of ex vivo lungs excised from the PF mice and the controls. (d) H&E histology and Masson's trichrome staining showing collagen deposition and alveolar damage in the PF mice; bar = 100 μm.

It has been reported that vascular dysregulation and abnormality are also present in chronic pulmonary disease such as PF.^25^ To investigate whether the developed in vivo quantitative imaging method could detect vascular hyperpermeability in chronic lung injuries, a bleomycin‐induced PF mouse model was used.^26^ At 50 days post‐repetitive bleomycin challenges, mice were imaged in vivo as described in the previous section. Representative 3D MPI‐CT cross‐sections of the PF model are shown in Figure 5a. Fibrotic lung regions can readily be identified in the CT images (solid arrows). The MPI image reveals patchy regions with vascular leakage, which can be identified from the hotspot in the color image (dashed arrows). Hyperpermeable regions partially matched the fibrotic regions, while some radiographically normal regions also exhibited higher permeability, which agrees with previously reported findings.^11^ The in vivo SEI of the bleomycin‐induced PF mice was 0.092 ± 0.029 cm^−3^ compared with 0.025 ± 0.003 cm^−3^ in the controls (*p* < 0.05) (Figure 5b). Ex vivo 2D MPI images confirmed the signal enhancement in the lungs of PF mice compared with the control mice (Figure 5c). In addition to MPI‐CT imaging, histopathological evaluations were performed to confirm the fibrotic changes. H&E and Masson's trichrome staining revealed characteristic alveolar damage with patchy areas of cellular consolidation and deposition of mature collagen, validating the establishment of PF (Figure 5d). Consistent with this, the Evans blue dye uptake was also increased in PF mice (Figure S1). Although the vascular injury and hemorrhage in the PF model were not as severe as those observed in the acute lung injury model, in vivo MPI imaging sensitively detected heterogeneous pulmonary vascular leakage under fibrosis, further validating the use of this technique to monitor vascular injury or dysfunction in chronic pulmonary diseases.

## 3 DISCUSSION

In this study, we demonstrated that 3D MPI‐CT successfully tracked pulmonary vascular disruption in vivo in both acutely injured and fibrotic mouse lungs. This method allows non‐invasive three‐dimensional visualization of pulmonary vascular leakage in living animals. In addition, image analysis quantitatively evaluated the SPIO extravasation, providing a surrogate measurement of pulmonary vascular permeability, which currently relies on ex vivo assays.

We first examined the feasibility of the proposed imaging method in an OA‐mediated acute lung injury model, which is known to induce endothelial damage and vascular permeability changes.^24^ In this model, a significantly increased pulmonary MPI signal was observed, confirming the capability of synomag® to extravasate into the lung interstitium through damaged vessel areas. In addition, MPI is sufficiently sensitive to register signals from diffusely distributed synomag® particles. Owing to its quantitative nature, MPI signal intensity directly reflects the severity of vessel damage and reveals the spatially heterogeneous vascular leakage. Regions with high MPI signals are consistent with those with high CT numbers, confirming that MPI successfully identifies the hyperpermeable tissue area. Moreover, the SEI quantified from the in vivo 3D MPI correlated well with the vascular permeability evaluated through ex vivo assays, further demonstrating the utility of this method for quantitatively assessing the integrity of endothelial barriers.

In our study, we noticed that OA‐mediated acute lung injury could be very severe in some individuals in whom hemorrhage had already occurred instead of vessel hyperpermeability. Therefore, a PF model with comparatively milder vessel damage was introduced to further validate our imaging method. Increased pulmonary vascular permeability is a typical physiological feature of PF; however, only a few studies to date have demonstrated this through in vivo imaging. In this study, we successfully visualized hyperpermeable vessel areas in the PF model using 3D MPI‐CT imaging. The 3D MPI image shows a heterogeneously distributed pulmonary signal in the PF model, highlighting regions with vessel damage. Interestingly, MPI imaging revealed some hyperpermeable vessel areas in the non‐fibrotic regions (as indicated by CT), which agrees with the findings of a previous MR study.^11^ Further studies are underway to track the dynamic changes in vascular permeability during PF and investigate the causality between vessel damage and tissue fibrosis.

To the best of our knowledge, this is the first MPI study of pulmonary vascular permeability in vivo. MPI has previously been used to monitor perfusion, the mucociliary clearance, and the inhaled aerosol distribution in pulmonary imaging. Our work adds to the current applications of MPI and extends its use in pulmonary research. Other imaging techniques, including NIR and MR, have also been proposed to assess pulmonary vascular permeability.^11^, ^15^, ^27^ Owing to the limited tissue penetration of optical photons, NIR pulmonary imaging is often conducted in an invasive manner, where the chest cavity is exposed, which cannot be conducted repetitively and hinders longitudinal monitoring. A recent ICG NIR imaging study successfully tracked lung vascular permeability noninvasively in rats, but it did not present 3D visualization capability.^15^ Indeed, as the optical signal rapidly deteriorates with increasing imaging depth, 3D NIR imaging of large animals can be challenging and has little clinical translational value. In comparison, MPI imaging registers the magnetic response from SPIOs that do not attenuate with imaging depth, which allows for 3D tomographic imaging in large animals and human. As tissue damage is often spatially heterogeneous, 3D visualization of vascular permeability provides the location and extent of barrier disruption, which is more informative than gross evaluation of vascular permeability across the entire lung. Gadofesovset‐based MR imaging has been reported to detect vascular leak in patients with IPF,^11^ thereby providing functional information that may be related to prognosis and disease progression. However, MR sensitivity is on the level of mM, while MPI sensitivity is several orders of magnitude higher.^28^ Therefore, MPI may be more suitable for detecting minute changes in pulmonary vascular permeability. In addition, human‐sized MPI systems are under development by several research groups^29^, ^30^ and SPIO‐based tracers such as ferumoxytol have been approved by FDA for human use. Therefore, MPI imaging shows great promise for monitoring lung injuries and hemorrhage in clinics. However, as a nanoparticle‐based technique, it may not be applicable to scenarios where vessels are only hyperpermeable to small molecules but not to macromolecules or nanoparticles.

A limitation of our current method is the animal pretreatment with clodronate liposomes to enhance the blood circulation and pulmonary delivery of the imaging tracers. Clodronate liposomes increase nanoparticle blood circulation time by partially depleting liver macrophages. This approach has been previously used to enhance nanoparticle delivery to tumor and fibrotic lungs to enhance therapeutic effects.^31^, ^32^ It has been reported that clodronate liposomes do not exhibit systematic toxicity and may even alleviate acute lung injuries.^31^, ^33^ However, the use of clodronate liposomes might affect the intravascular macrophage populations and may not be an ideal approach for future clinical translation. This problem can be resolved by constant infusion instead of bolus injection of the tracers. Surface‐modified long‐circulating tracers may also be used to enhance pulmonary delivery, as reported in several studies.^34^, ^35^, ^36^, ^37^ In fact, even in mice without clodronate liposome pretreatment, we observed increased SPIO accumulation in the injured lungs, but to a lesser extent (Figure S3). In cases of human imaging, it has been shown that nanoparticles have much longer blood circulation times in human than in mice,^34^ hence long circulating tracers may not be necessary in the end. Moreover, the current MPI scanner is designed for whole body imaging, a dedicated chest coil with higher sensitivity to pulmonary signals would be helpful for future use of this technique.

## 4 CONCLUSIONS

Overall, this study, for the first time, demonstrates the feasibility of using 3D MPI to sensitively visualize SPIO extravasation from the blood to the lung in living animals, thus providing a noninvasive and quantitative tool to assess pulmonary vascular leakage in vivo. 3D MPI shows great promise for pulmonary research and future clinical translation.

## 5 MATERIALS AND METHODS

### 5.1 Animals

Male Kunming mice (7–8 weeks old) and male C57BL/6J mice (7–8 weeks old) were purchased from Beijing Vital River Laboratory Animal Technology Co., Ltd. and acclimatized in a specific pathogen‐free (SPF) facility under controlled conditions for 7 days before use. In acute lung injury experiments, five independent sets of Kunming mice were used for in vivo MPI and CT imaging, ex vivo MPI, H&E histology, Prussian blue staining, and apoptosis assay, and three independent sets of mice were used for the BALF protein test and Evans blue extravasation test. In chronic lung injury experiments, three independent sets of C57BL/6J mice were used for in vivo MPI and CT, ex vivo MPI, H&E histology, Masson's trichrome staining, and Evans blue extravasation test. All animal experiments were conducted in accordance with institutional guidelines and approved by the Animal Ethics Committee at the Institute of Automation of the Chinese Academy of Sciences (IA21‐2203‐10).

### 5.2 Acute lung injury model

Oleic acid (OA, 112‐80‐1, TCI) was used to induce acute lung injury and immediate vascular permeability changes.^24^ Kunming mice were randomly assigned to experimental (OA) or control (CTR) groups. For mice in the OA group, 0.1 mL/kg OA (in 40 μL ethanol) was injected into the tail vein to induce acute lung injury, while the mice in the CTR group were administered 40 μL of vehicle. Arterial oxygen saturation (SpO_2_) was monitored by wrapping a pediatric pulse oximeter (60D, Heal Force Bio‐Meditech Holdings Limited, Shanghai, China) around the neck of the mice. Immediately after administration of OA or vehicle, 3D MPI and CT imaging were performed in vivo. At the end of the imaging experiment, the mice were euthanized and lung tissue was extracted for ex vivo 2D MPI, histopathology, Prussian Blue staining, and apoptosis assay.

### 5.3 Chronic lung injury model

A bleomycin (BLM)‐induced PF model was used for the chronic lung injury experiments.^25^, ^26^ C57BL/6J mice were randomly assigned to experimental (PF) or control (CTR) groups. The mice in the PF group were anesthetized by intraperitoneal injection of a mixture of 1.25% tribromoethanol (T48402, Sigma‐Aldrich) and 2.5% 2‐methyl‐2‐butanol (152463, Sigma‐Aldrich), and were hung on an intubation platform. A small animal laryngoscope (LS‐2‐C, Penn‐Century) was used to facilitate visualization of the trachea and epiglottis. Fifty microliters of 1 mg/mL BLM (HY‐17565, MedChemExpress) was administered intratracheally using a MicroSprayer® Aerosolizer (YAN 30012, Yuyan Instruments Co, Ltd., Shanghai, China). For the mice in the CTR group, 50 μL of vehicle (phosphate‐buffered saline, PBS) was administered. This procedure was repeated four times, on days 0, 12, 24, and 36. On day 50, MPI and CT were performed in vivo. The mice were euthanized after the imaging experiments and the lung tissue was extracted for ex vivo MPI and histological assessment of fibrosis.

### 5.4 SPIO characterization

For the imaging experiments, a commercially available dextran coated SPIO synomag® (103‐02‐301, micromod Partikeltechnologie GmbH) was used as the tracer. The core size and morphology of the synomag® were examined using transmission electron microscopy (TEM, JEM 1200EX, Jeol, Tokyo, Japan). The hydrodynamic size was measured using a Dynamic Light Scattering Zetasizer (Nano‐ZS90, Malvern Instruments, Malvern, UK).

### 5.5 In vivo 3D MPI and CT imaging

To enhance the pulmonary delivery efficiency of synomag® and maximize the pulmonary signal, a previously reported nanoparticle long‐circulation technique was adopted.^31^ Briefly, mice were intravenously injected with 200 μL of clodronate liposomes (C‐005, Liposoma BV, The Netherlands) 24 h before imaging. On the day of imaging, the synomag® particles were diluted with PBS and sonicated for 5 min prior to administration. Prior to imaging, mice were intravenously injected with 3 mg Fe/kg synomag® particles. At 8 h post‐tracer administration, mice were anesthetized and 3D MPI images of the mice were acquired using the default scan mode and 35 projections using the MOMENTUM scanner (Magnetic Insight, CA, USA). Three fiducials, each containing 2 μL of synomag® stock solution, were placed on the animal bed as landmarks and imaged together with the animal. After 3D MPI imaging, the animal and animal bed were scanned using our in‐house built micro‐CT scanner. CT scans were performed using the following parameters: number of projections, 288; X‐ray tube voltage = 90 kV; X‐ray tube current = 88 μA; FOV, 10 cm; voxel size, 144 μm. 3D MPI and CT images were co‐registered based on the fiducials in the 3Dmed software using the CT and MPI Visualization Tools (http://www.radiomics.net.cn/platform/version/3). After in vivo imaging, the mice were euthanized and the major organs were extracted. 2D ex vivo MPI images of the lung, liver, spleen, heart, and kidney were acquired using isotropic scan mode.

### 5.6 Quantitative image analysis

To perform quantitative analysis of the 3D images, the lung region of interest (ROI) was manually selected based on the anatomical structure in each slice of the CT image. The ROIs were applied to the co‐registered MPI‐CT images and the MPI signal intensity of each pixel in the lung ROIs was extracted. We defined the SPIO extravasation index (SEI) as the pixel intensities (PXLI) summed over all the pixels in the lung divided by PXLI summed over the entire body and normalized by the volume of the lung ($V_{\text{lung}}$, cm^3^):

(1) $$\mathrm{SEI}=\frac{\sum_{\text{lung}}{\text{PXLI}}_{i,j,k}}{\sum_{\text{whole body}}{\text{PXLI}}_{i,j,k}*V_{\text{lung}}},$$

where i,j,k denote the coordinates that identify the location of each pixel. 3D image segmentation and quantification were performed using the CT & MPI Visualization Tools in 3Dmed software. 2D image quantification and processing were performed using VivoQuant™ software (VivoQuant 4.0, Invicro, Boston, MA, USA).

### 5.7 Evans blue extravasation test

The Evans blue extravasation test was used as the gold standard for pulmonary vascular permeability assessment. A 2% Evans blue (Macklin, E808783) saline solution was prepared, and the tail vein was injected (20 mg/kg).^38^ At 30 min post‐injection, the mice were anesthetized by intraperitoneal injection of a mixture of 1.25% tribromoethanol and 2.5% 2‐methyl‐2‐butanol. The chest cavity was exposed, and the abdominal aorta was excised. To remove the peripheral blood and dye from the cardiopulmonary circulation, a small cut was made on the left atrium, and 200 mL of PBS was gently perfused through the apex of the right ventricle. Lung tissue was collected, homogenized, and incubated in formamide at 37°C for 48 h. The homogenates were centrifuged (8000 rpm, 10 min), and the supernatants were collected. Absorption at 630 nm was measured using a microplate reader (PerkinElmer Victor Nivo), and the tissue concentration of Evans blue was calculated based on the standard curve calibrated with Evans blue formamide solutions (Figure S2).

### 5.8 BALF protein test

To collect BALF, the mice were anesthetized by intraperitoneal injection of a mixture of 1.25% tribromoethanol and 2.5% 2‐methyl‐2‐butanol and hung on the intubation platform. BALF was obtained by cannulating the trachea with a 20‐gauge needle and infusing the lungs three times with sterile PBS (0.5 mL).^39^ The recovered BALF was centrifuged at 1350 rpm for 5 min at 4°C and the supernatant was immediately transferred to a −80°C freezer. The total protein concentration in the BALF was measured using a BCA Protein Assay Kit (Servicebio, IG2026‐1000 T), as instructed.

### 5.9 Histopathological evaluation

At the end of the imaging experiments, the mice were euthanized and the lungs were harvested. The lungs were immersed in a 4% paraformaldehyde fix solution (P0099, Beyotime Biotechnology, Shanghai, China) and embedded in paraffin. Whole lobes of the lungs were cut into 5 μm‐thick sections and stained with H&E. To assess collagen disposition in PF models, Masson's trichrome staining was carried out according to the manufacturer's instructions (G1346, Solarbio Science & Technology Co., Ltd., Beijing, China). Extravasation of SPIOs in the lung tissue and alveolar space was evaluated by Prussian blue staining (G1424, Solarbio Science & Technology Co., Ltd.). Apoptosis assays were performed using terminal deoxynucleotidyl transferase biotin‐dUTP nick‐end labeling (TUNEL, Servicebio, G1504) staining. After TUNEL staining, the tissue sections were washed with PBS and incubated with DAPI for nuclear staining. All histopathological images were acquired using a Pannoramic MIDI scanner (3DHISTECH Ltd.).

### 5.10 Statistical analysis

All data are presented as means ± the standard deviation (SD). The statistical analyses were performed using Prism ver.9 (GraphPad Software, Boston, MA, USA). The Mann–Whitney *U* test was used for statistical evaluations.^40^ *p* < 0.05 was considered statistically significant. Significance was denoted by **<0.01, *<0.05.

## AUTHOR CONTRIBUTIONS

**Xin Feng:** Conceptualization (lead); data curation (equal); funding acquisition (equal); investigation (lead); methodology (lead); project administration (equal); visualization (equal); writing – original draft (lead); writing – review and editing (equal). **Pengli Gao:** Data curation (equal); investigation (equal); methodology (equal); validation (equal); visualization (equal); writing – review and editing (equal). **Yabin Li:** Data curation (equal); investigation (equal); writing – review and editing (equal). **Hui Hui:** Funding acquisition (equal); project administration (equal); software (lead); supervision (equal); writing – review and editing (equal). **Jingying Jiang:** Funding acquisition (equal); project administration (equal); resources (equal); supervision (equal); writing – review and editing (equal). **Fei Xie:** Project administration (equal); supervision (equal); validation (equal). **Jie Tian:** Funding acquisition (lead); project administration (lead); resources (lead); supervision (lead); writing – review and editing (equal).

## FUNDING INFORMATION

This work was supported in part by the National Natural Science Foundation of China (Grant Nos. 62027901, 81227901, 81930053, 82302407, 61976223 and 81971662), the Natural Science Foundation of Beijing City (Grant Nos. 7232346, 7202105), and the Medical Program (BLB20J002). The authors would like to acknowledge the instrumental and technical support of Multimodal Biomedical Imaging Experimental Platform, Institute of Automation, Chinese Academy of Sciences.

## CONFLICT OF INTEREST STATEMENT

The authors have no conflicts to disclose.

### PEER REVIEW

The peer review history for this article is available at https://www.webofscience.com/api/gateway/wos/peer‐review/10.1002/btm2.10626.

## Supporting information


**Appendix S1.** Supporting information

**FIGURE S1.** Standard absorbance curve of Evans blue solution.

**FIGURE S2.** Pulmonary vascular permeability assessment by Evans blue extravasation test.

**FIGURE S3.** Pulmonary SPIO accumulation in OA‐treated mice without clodronate liposome treatment. (a) Representative 3D MPI‐CT images of the OA‐treated mice (OA) and the controls (CTR) without clodronate liposome treatment. (b) SEI of OA‐treated (*n* = 4) and control mice (*n* = 4) quantified from in vivo 3D MPI images. *p < 0.05.

## Data Availability

The data that support the findings of this study are available from the corresponding author upon reasonable request.
