# Supplementary figures and images for: First magnetic particle imaging to assess pulmonary vascular leakage in vivo in the acutely injured and fibrotic lung
Source: Bioeng Transl Med. 2023 Nov 29;9(2):e10626. doi: 10.1002/btm2.10626 (PMC10905553; doi:10.1002/btm2.10626)

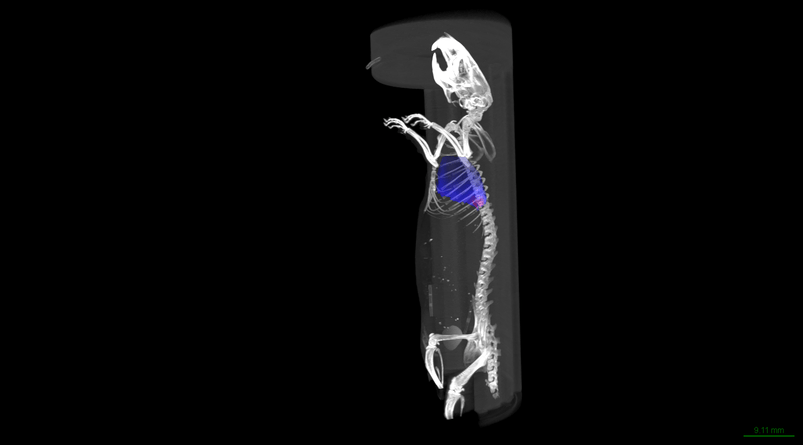

Supplement: Supplementary file 1 — Appendix S1. Supporting information [file BTM2-9-e10626-s004.zip › MPI-CT-CTR group.gif]

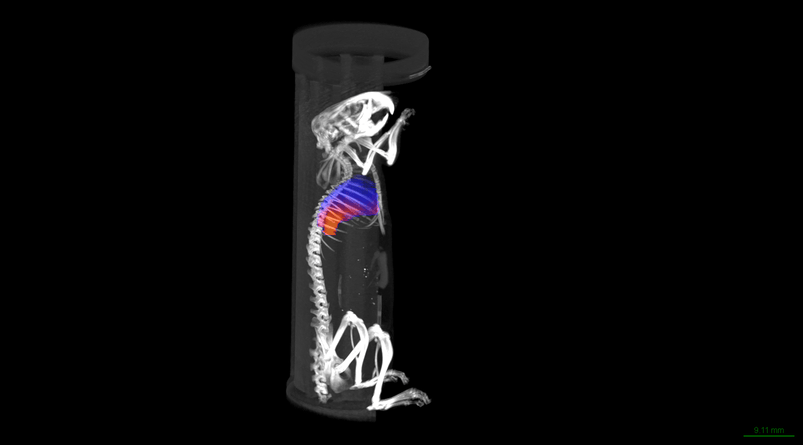

Supplement: Supplementary file 1 — Appendix S1. Supporting information [file BTM2-9-e10626-s004.zip › MPI-CT-OA group.gif]

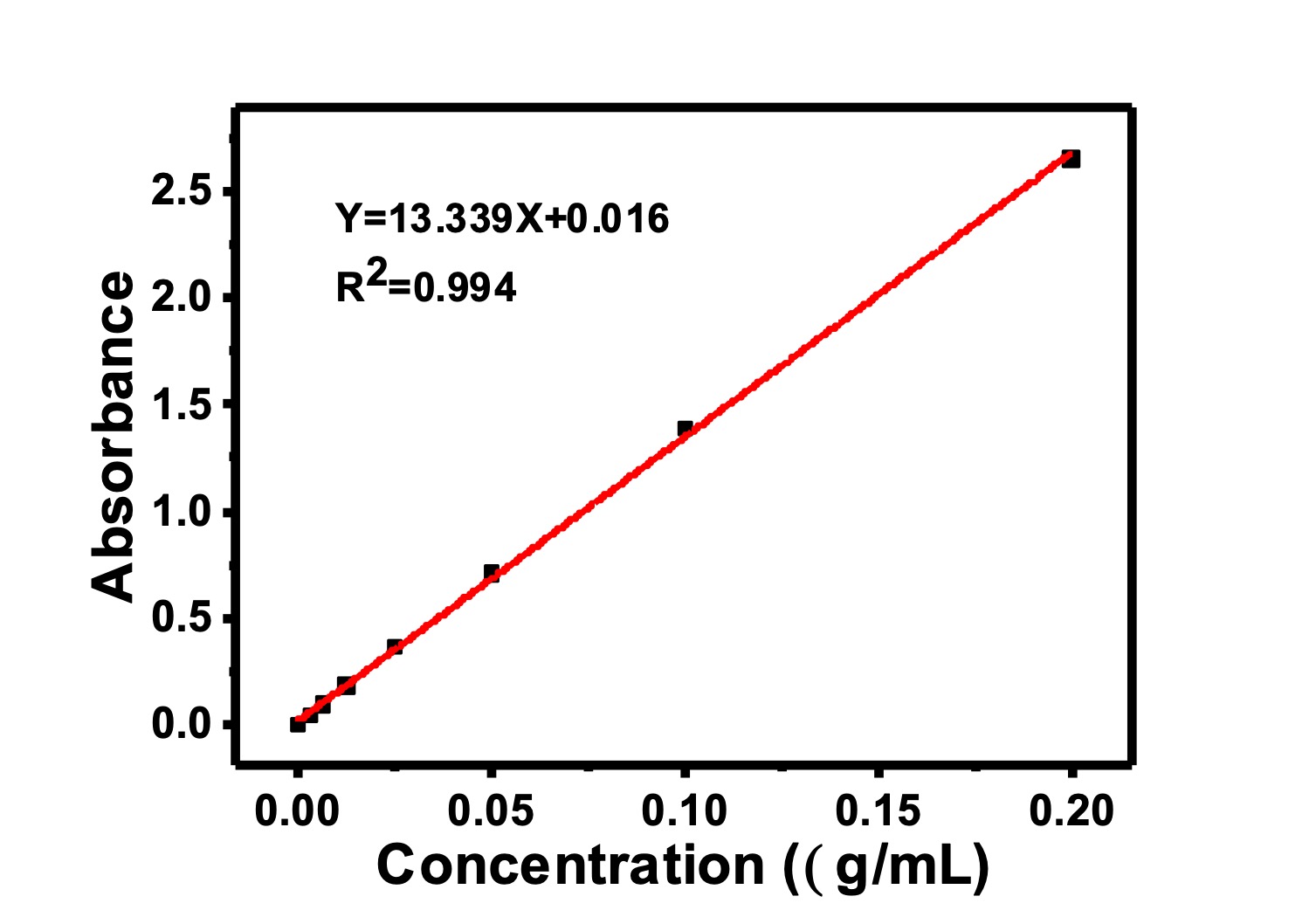

Supplement: Supplementary file 2 — FIGURE S1. Standard absorbance curve of Evans blue solution. [file BTM2-9-e10626-s003.jpg]

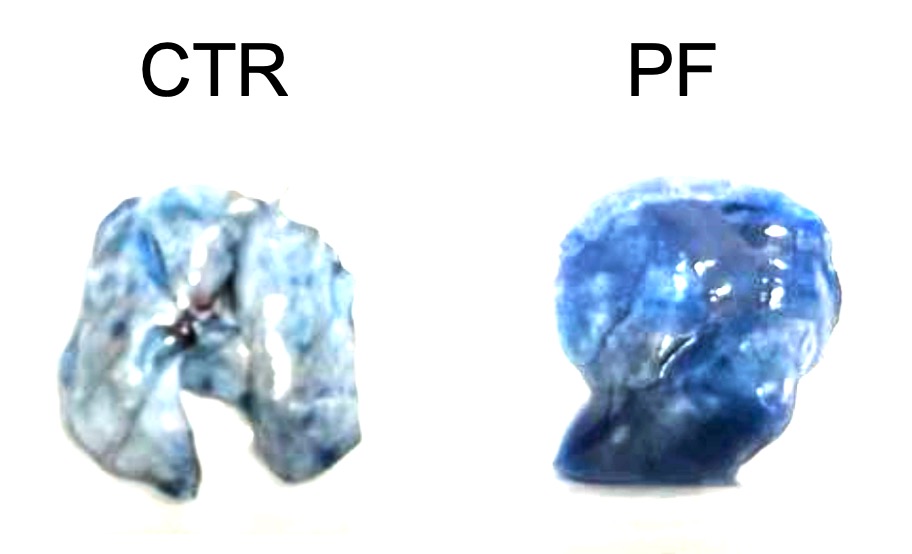

Supplement: Supplementary file 3 — FIGURE S2. Pulmonary vascular permeability assessment by Evans blue extravasation test. [file BTM2-9-e10626-s002.jpg]

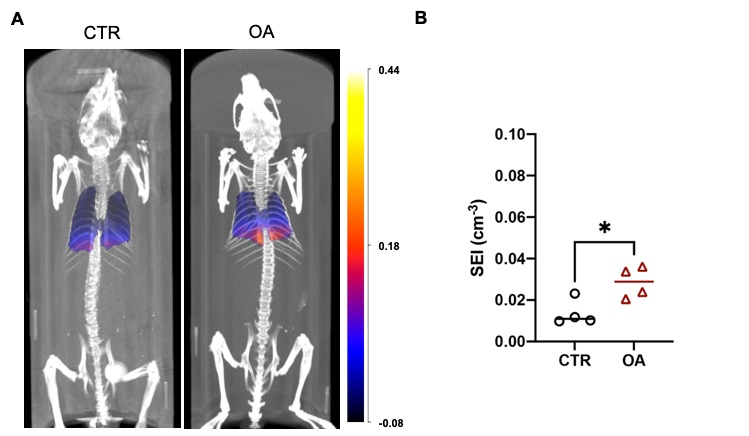

Supplement: Supplementary file 4 — FIGURE S3. Pulmonary SPIO accumulation in OA‐treated mice without clodronate liposome treatment. (a) Representative 3D MPI‐CT images of the OA‐treated mice (OA) and the controls (CTR) without clodronate liposome treatment. (b) SEI of OA‐treated (n = 4) and control mice (n = 4) quantified from in vivo 3D MPI images. *p < 0.05. [file BTM2-9-e10626-s001.jpg]
